# Glymphatic dysfunction assessed by DTI-ALPS index predicts early cognitive impairment in acute subcortical infarcts: a prospective clinical cohort study

**DOI:** 10.3389/fneur.2025.1605889

**Published:** 2025-07-09

**Authors:** Yirong Wang, Mei Yang, Xingmao Zeng, Shuai Wang, Wenmin Zhang, Weidong Wang, Yang Du, Jurong Ding, Xin Ding

**Affiliations:** ^1^Department of Neurology, Chengdu Second People’s Hospital, Chengdu, China; ^2^School of Automation and Information Engineering, Sichuan University of Science and Engineering, Zigong, China; ^3^Department of Neurology, Chengdu Fifth People's Hospital, Chengdu, Sichuan, China; ^4^Department of Outpatient, General Hospital of Western Theater Command, Chengdu, Sichuan, China

**Keywords:** glymphatic system, DTI-ALPS, subcortical infarction, cognitive impairment, diffusion tensor imaging

## Abstract

**Background:**

The glymphatic system (GS), responsible for clearing neurotoxic proteins (such as β-amyloid and tau protein), is critical in stroke pathophysiology. However, its role in acute post-stroke cognitive impairment (PSCI) remains unclear. We investigated GS dysfunction via the DTI-ALPS index in acute subcortical infarct patients and its association with early cognitive decline.

**Methods:**

This prospective cohort included 29 subcortical infarct patients and 25 healthy controls (HC). Participants underwent 3.0 T MRI (DTI/structural sequences) and Montreal Cognitive Assessment (MoCA) at 7 and 90 days post-stroke. Bilateral DTI-ALPS indices were calculated. Group comparisons and Spearman correlations were analyzed.

**Results:**

The DTI-ALPS index of the lesion (1.371 ± 0.170) and non-lesion side (1.310 ± 0.198) in the SI group were significantly lower than that in HC group (1.568 ± 0.115) (both of *p* < 0.001, respectively). While, the DTI-ALPS index of the lesion side was no significant difference than that of the non-lesion side in subcortical infarct group (*p* = 0.214). The scores of MoCA in 7 days and 90 days after stroke were significantly lower than those in HC group (*p* < 0.001). In patients with subcortical infarct, MoCA scores at 7 days showed significant correlation with lesion-side DTI-ALPS index (*r* = 0.510, *p* = 0.005) but not with non-lesion DTI-ALPS values (*r* = 0.174, *p* = 0.259). Notably, we observed significant correlations between MoCA scores at 90 days post-stroke and DTI-ALPS index, which were consistently demonstrated in the lesion, non-lesion, and mean bilateral measurements. The ROC analysis demonstrated that the DTI-ALPS index showed moderate discriminative ability (AUC = 0.868) in differentiating patients with cognitive impairment from those with normal cognition following subcortical infarction, exhibiting excellent sensitivity (96.0%) but suboptimal specificity (65.5%).

**Conclusion:**

Ischemic stroke leads to glymphatic dysfunction, which is associated with early post-stroke cognitive impairment.

## 1 Introduction

Post-stroke cognitive impairment (PSCI) represents a devastating consequence of cerebral infarction (1), with its severity closely associated with lesion topography while demonstrating substantial heterogeneity in cognitive outcomes based on infarct location. Strategic infarctions in the left frontotemporal lobes, thalamus, and right parietal lobe exhibit particularly strong correlations with PSCI development (2).

Cerebral infarction triggers cytotoxic/vasogenic edema, neuroinflammation, and blood–brain barrier disruption (3), which collectively promote secondary neurodegeneration and cognitive decline. The pathological cascade following stroke, including impaired clearance of metabolic byproducts such as β-amyloid (Aβ) (4), tau protein (5), lactate (6) and iron (7), contributes significantly to PSCI pathogenesis, though the underlying mechanisms remain incompletely understood. In recent years, further explore pathophysiological mechanisms of PSCI based on the rise of neuroimaging research (8, 9), may facilitate the acquisition of imaging markers of PSCI and the development of novel treatments for restoring cognitive decline.

Known as the glymphatic system (GS), a pathway for drainage and exchange between cerebrospinal fluid (CSF) and interstitial fluid (ISF). CSF flows into the brain parenchyma along the perivascular spaces of arteries and exchanges with ISF in the GS, subsequently draining out along the perivascular spaces of veins (10). Available studies have found that the glymphatic system affects the pathological changes and outcomes of stroke (11). Based on studies in animal models of post-stroke dementia, chronic cerebral hypoperfusion impairs glymphatic system function, reduces clearance of tau protein from the brain interstitial fluid, and triggers its neuropathological aggregation in peri-infarct regions (5). This demonstrates that impaired glymphatic clearance serves as a critical link connecting post-stroke vascular injury to pathological tau accumulation. Further studies are needed to confirm the relationship between the glymphatic system and PSCI. Treatment strategies to improve the clearance of waste removal may also be a promising approach to improve cognitive impairment and the prevention of PSCI.

Diffusion tensor imaging analysis along the perivascular space (DTI-ALPS) is a quantitative assessment of the function of lymphatic flow and clearance along the perivascular space (PVS) (12). A successful application in the exploring the pathophysiology of Alzheimer’s disease (AD) (13), Parkinson’s disease (PD) (14), headache (15) has been reported. Some reported that DTI-ALPS showed lower values in ischemic stroke, suggesting impaired lymphatic function. After the initial injury, the DTI-ALPS index increased with time after stroke onset, suggesting a recovery of lymphatic function (16). Another study found that the DTI-ALPS index was independently positively associated with episodic memory in cerebral small vessel disease (CSVD) patients and was an early potential marker for recognition of cerebral infarction (17). PSCI affects 35–47% of survivors, yet its pathophysiological mechanisms remain elusive (18). Emerging evidence implicates glymphatic dysfunction in neurodegenerative protein clearance (19, 20), but its role in acute stroke-related cognitive decline is poorly understood. We hypothesize that DTI-ALPS, a novel imaging biomarker of glymphatic activity, may bridge this gap.

Cortical infarctions (involving the frontal, temporal, or parietal lobes) can directly impair cognitive domains. Additionally, due to their typically larger lesion volumes, cortical infarcts may exert mass effects that compress perivascular spaces, thereby impairing glymphatic clearance (21, 22). This mechanism could exacerbate post-stroke cognitive dysfunction independent of direct neuronal damage. To reduce confounding bia, we selected patients with subcortical infarction. In this study, to quantitatively investigate the pathological characteristics of GS in subcortical infarct patients and the relationship with cognitive impairment by non-invasive DTI analysis method, we consider that glymphatic dysfunction after subcortical infarct may be related to early cognitive dysfunction.

## 2 Materials and methods

### 2.1 Study population and sample size

**Figure 1:**
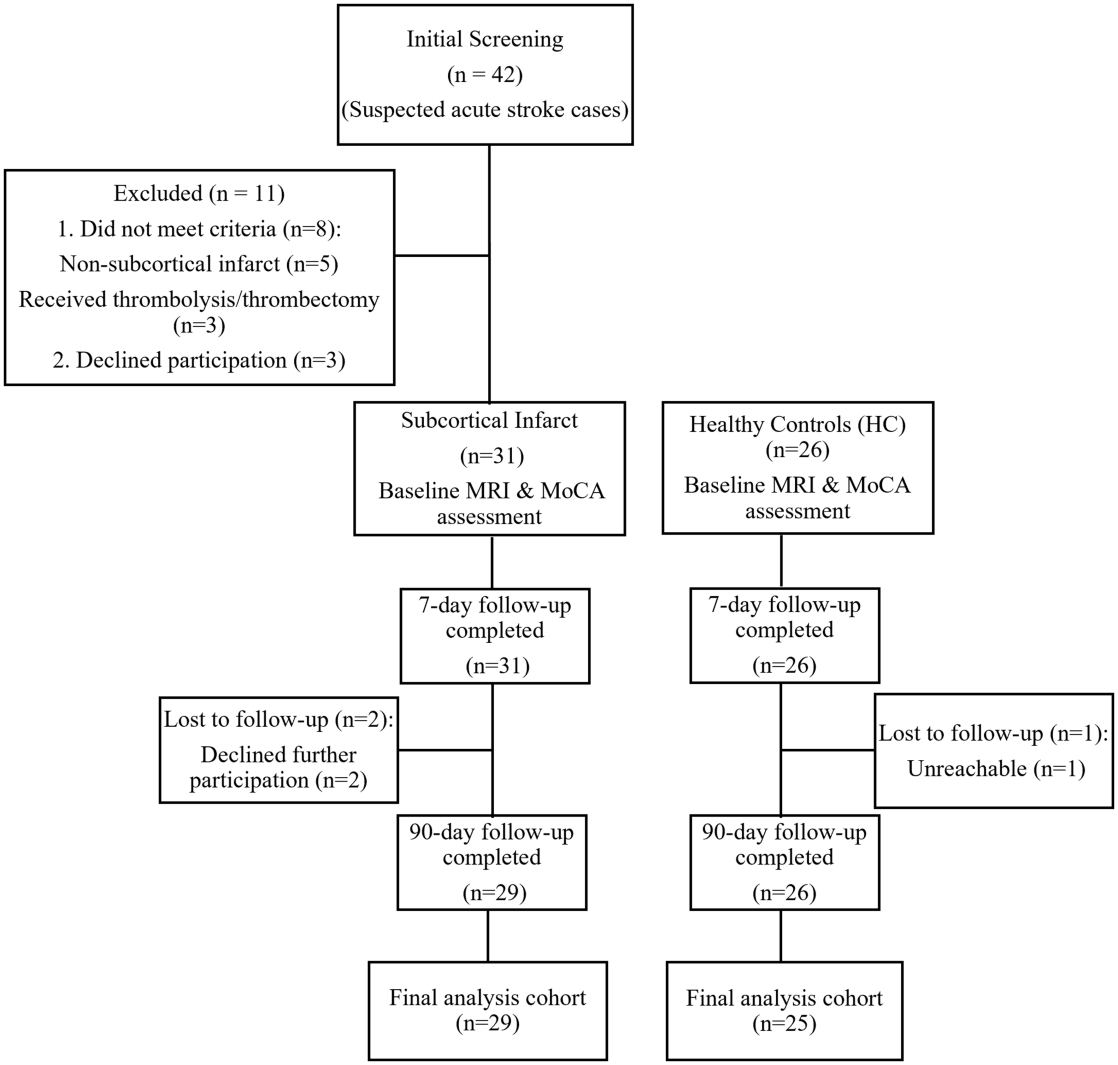
Flowchart of participant inclusion, exclusion, and analysis.

The sample size was calculated using G*Power 3.1, assuming a medium effect size (Cohen’s *d* = 0.8) for glymphatic dysfunction (DTI-ALPS index) between stroke patients and healthy controls (HC), based on prior literature (16). From 42 initially screened acute stroke patients, 11 were excluded (Figure 1): 8 did not meet inclusion criteria (included 5 non-subcortical infarcts and 3 received thrombolysis/thrombectomy) and 3 declined participate. Follow up of 31 eligible subcortical infarct patients, 2 were lost to follow-up (1 relocation; 1 withdrawal), leaving 29 for final analysis. Twenty-six age−/sex-matched HC were recruited, with 1 lost to follow-up (unreachable), resulting in 25 for analysis. Inclusion criteria for SI patients were (1) aged ≥18 years; (2) MRI-confirmed acute subcortical infarct (within 72 h from onset) with small infarcts (<15 mm diameter) to minimize perilesional edema, exclusively involving the basal ganglia/periventricular. No thrombolysis/thrombectomy; (3) Right-handedness pre-stroke. HC met the following criteria: (1) age being 18 years or older; (2) right-handedness. Exclusion (all): (1) Recurrent stroke; (2) neurodegenerative diseases; (3) MRI contraindications. This study was approved by the Ethics Committee of Chengdu Second People’s Hospital (No. 2023491). Written informed consent was obtained.

### 2.2 Clinical data collection

Demographic and clinical data were collected retrospectively, including age, sex, education level, history of hypertension, coronary artery disease, diabetes, atrial fibrillation, smoking, and alcohol consumption. Lesion characteristics and National Institutes of Health Stroke Scale (NIHSS) scores were recorded. Cognitive function was assessed using the Montreal Cognitive Assessment (MoCA) at 7 and 90 days post-stroke. The MoCA evaluates domains including attention and concentration, executive functions, memory, language, visuospatial abilities, abstract thinking, calculation, and orientation (23). Two blinded experts (a neurologist and a radiologist) independently analyzed MRI data to confirm lesion location.

### 2.3 Imaging acquisition

All participants underwent brain MRI using a 3.0 T Ingenia scanner (Philips) with a 32-channel head coil. Clinical routine diffusion-weighted imaging (DWI) reveals the location of focus in patients with subcortical infarct. DWI scans obtained with repetition time (TR) = 5,000 ms, echo time (TE) = 84 ms, field of view (FVO) = 24 × 24 mm, matrix size = 128 × 128, slice thickness = 5 mm. A DTI data was obtained using the following parameters: TR = 4,934 ms, TE = 88 ms, matrix = 112 × 112 mm^2^, FOV = 224 × 224 mm^2^, number of diffusion gradient directions = 32, b value = 1,000 s/mm^2^, isotropic voxel = 2 mm × 2 mm × 2 mm. slice thickness = 2 mm, number of slices = 74.

### 2.4 DTI-ALPS index calculation

The DTI-ALPS method quantifies glymphatic system activity by measuring multidirectional diffusivity maps (12, 24). An ALPS index approaching 1 indicates either minimal perivascular water diffusion influence or severe glymphatic dysfunction. Higher index values reflect better glymphatic system functionality.

**Figure 2:**
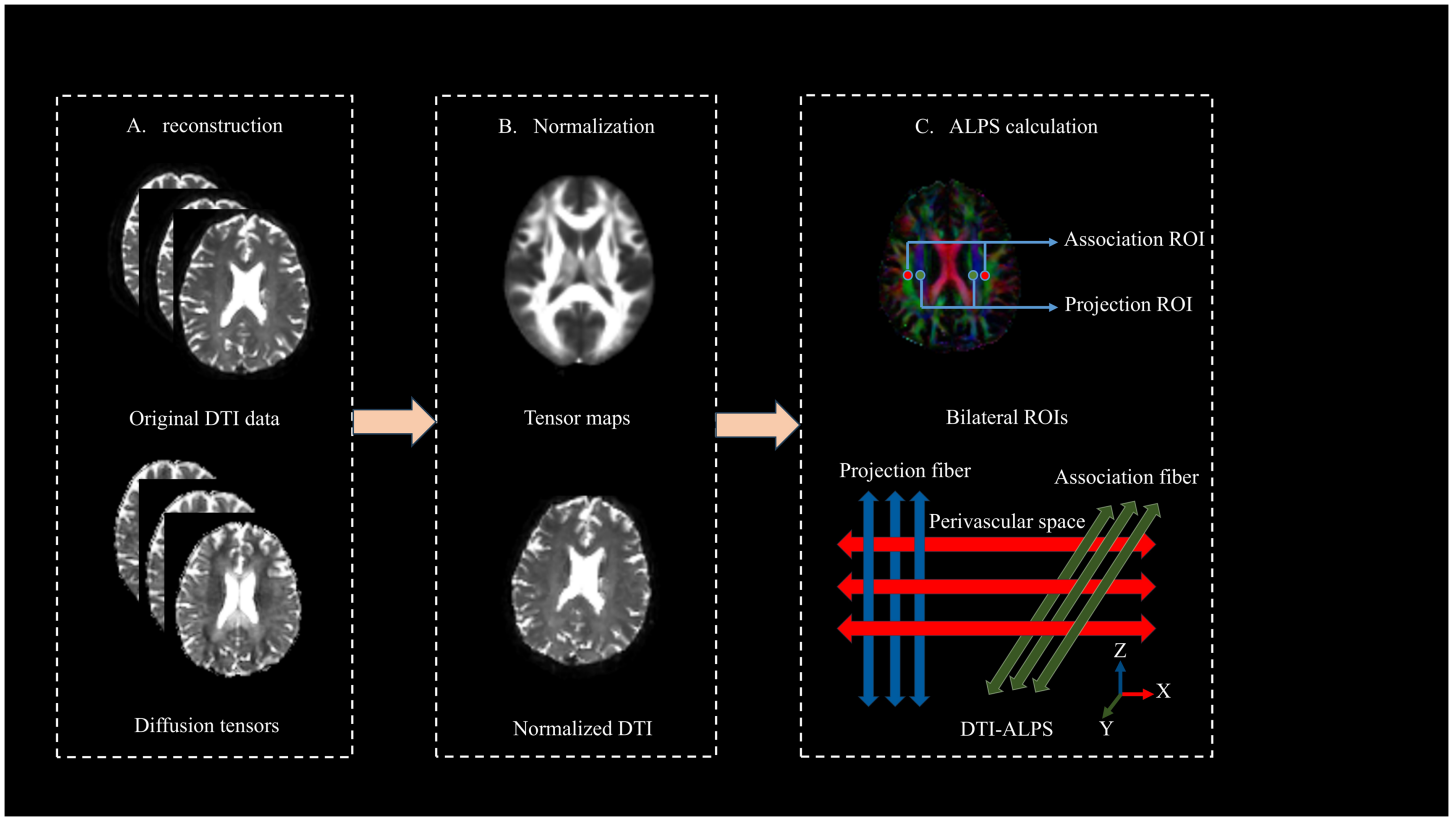
Computational workflow for deriving DTI-ALPS index from raw DTI data.

The DTI-ALPS pre-processing through a three-stage pipeline in MATLAB R2019b (MathWorks) using FSL 6.0 (FMRIB Software Library) illustrated in Figure 2. First, raw DTI data underwent format conversion, eddy current correction, motion correction, and tensor fitting to generate diffusion metrics. At the lateral ventricle level, two 5-mm spherical regions of interest (ROIs) with voxel size-matched to 1.5 × 1.5 × 1.5 mm^3^ were manually positioned by a certified neuroradiologist in projection and association fiber territories of bilateral hemispheres, adhering to strict anatomical criteria that required ROI placement adjacent to medullary veins, while rigorously avoiding cerebral infarct zones via DWI-FLAIR co-registration. Subsequently, individual datasets were normalized to MNI152 standard space. For each subject, voxel-wise tensor calculations generate parametric maps, including color-coded fractional anisotropy (FA) maps and three diffusivity maps (along the *x*-, *y*-, and *z*-axes). Projection fibers (dominant along the *z*-axis) and association fibers (dominant along the *y*-axis) are visualized in blue and green, respectively.

The DTI-ALPS index is defined as follows:


$$\mathrm{DTI}-\text{ALPS index}=\frac{\text{Mean}(\text{Dxproj},\text{Dxassoc}\;)}{\text{Mean}(\text{Dyproj},\text{Dzassoc}\;)\;}$$

The calculation of the DTI-ALPS index is based on the ratio of water molecule diffusion capacities in specific directions. At the level of lateral ventricles, the ALPS index as the ratio of mean of diffusivity along the *x*-axis in the projection fibers (Dxproj) and *x*-axis in the association fibers (Dxassoc) to the mean of diffusivity along the *y*-axis in the projection fibers (Dxproj) and *z*-axis in the association fibers (Dzassoc).

### 2.5 Statistical analysis

Data were analyzed using SPSS version 25.0. Kolmogorov–Smirnov tests were used to test for normal distribution. Continuous variables are expressed as mean ± standard deviation; categorical variables as frequency (%). Normality was assessed with Kolmogorov–Smirnov tests. Group differences in MoCA scores were analyzed with analysis of covariance (ANCOVA) with adjusting for education years and vascular risk factors (hypertension, diabetes, atrial fibrillation). ANCOVA (adjusting for sex, age, and body weight) compared DTI-ALPS indices between groups. Spearman’s partial correlations analysis evaluated associations between MoCA scores and DTI-ALPS indices. A two-tailed *p* < 0.05 was considered statistically significant.

## 3 Results

### 3.1 Comparison of clinical data

Twenty-nine subcortical infarct patients (16 males and 13 females) with a mean age of 65.35 ± 11.11 and 25 HC participants (12 males and 13 females) with a mean age of 63.88 ± 7.76 were enrolled in this study. No significant differences in age and gender were demonstrated between healthy controls and subcortical infarct patients (*p* > 0.05). After adjusting for education year and vascular risk factors (hypertension, diabetes, atrial fibrillation), subcortical infarct patients demonstrated significantly lower MoCA scores at 7 days and 90 days post stroke compared to HC group (*p* < 0.001) (Table 1).

**Table 1:** Clinical characteristics of subcortical infarction patients and healthy controls.

Category	Subcortical Infarction (*n* = 29)	HC (*n* = 25)	*χ*^2^/*t-*value/*F-*value	*p*
Age (years)	65.35 ± 11.11	63.88 ± 7.76	0.567	0.573
Gender (Male %)	16 (66%)	12 (48%)	0.277	0.785
Onset time (H)	36 (6, 70)	–	–	–
Diabetes	6 (20%)	5 (20%)	0.461	0.730
Hypertension	12 (41%)	13 (52%)	0.122	0.774
Atrial fibrillation	2 (6%)	0		
Coronary artery disease	1 (3%)	0		
^*^Adjusted MoCA score of 7 days post-stroke	17.994 ± 0.762	28.647 ± 0.828	79.296	**<0.001**
^*^Adjusted MoCA score of 90 days post stroke	19.079 ± 0.726	28.709 ± 0.790	71.205	**<0.001**

^*^Adjusted for education years, hypertension, diabetes, atrial fibrillation. Bold values < 0.001, differences are statistically significant.

### 3.2 DTI-ALPS index group differences

**Figure 3:**
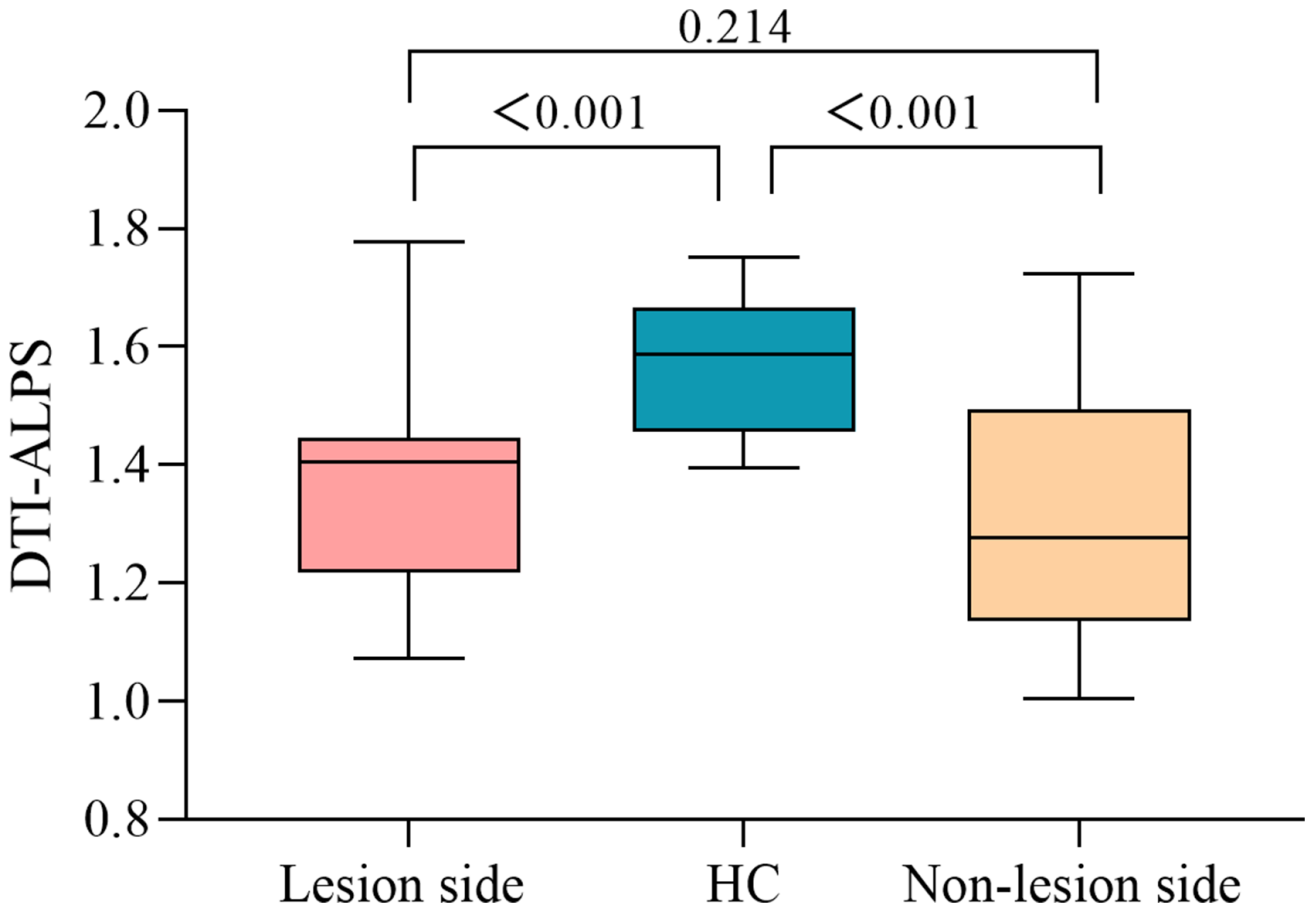
DTI-ALPS index differences between subcortical infarction patients and healthy controls.

DTI-APLS of differences between subcortical infarct and HC group were statistically significant (*p* < 0.05). A significant lower DTI-ALPS index in the lesion (1.371 ± 0.170) and non-lesion side (1.310 ± 0.198) were found in the subcortical infarct group compared to the HC group (1.568 ± 0.115) (*p* < 0.001, respectively) While, the DTI-ALPS index of the lesion side was no significantly than that of the non-lesion side in subcortical infarct group (*p* = 0.214) (Figure 3).

### 3.3 Associations between MoCA score and the DTI-ALPS index of subcortical infarct patients

**Figure 4:**
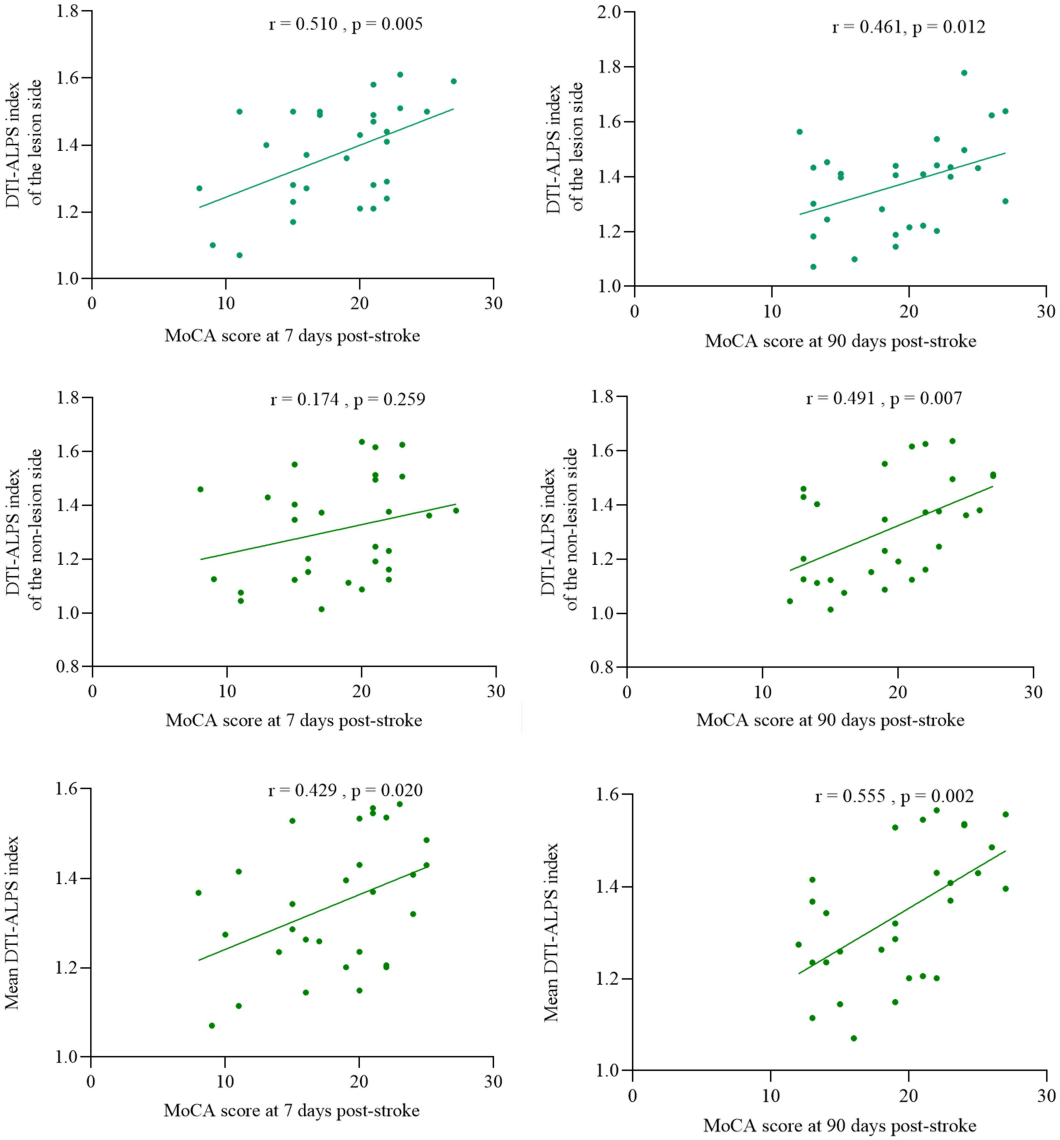
Association of DTI-ALPS index with cognitive function at 7 and 90 days post-stroke.

After adjusting for education and cerebrovascular risk factors (hypertension, diabetes, and atrial fibrillation), partial correlation analysis revealed the ALPS index of lesion side showed significant positive correlations with MoCA score both 7 days (*r* = 0.510, *p* = 0.005) and 90 days after stroke (*r* = 0.461, *p* = 0.012). The ALPS index of non-lesion side demonstrated a positive correlation with MoCA scores after stroke at 90 days (*r* = 0.491, *p* = 0.007) but no significant association with 7 days scores (*r* = 0.174, *p* = 0.259); The mean bilateral ALPS index positively correlated with both post stroke at 7 days (*r* = 0.429, *p* = 0.020) and 90 days MoCA scores (*r* = 0.555, *p* = 0.002) (Figure 4).

### 3.4 Predictive maker of DTI-ALPS index for cognitive impairment in subcortical infarction

**Figure 5:**
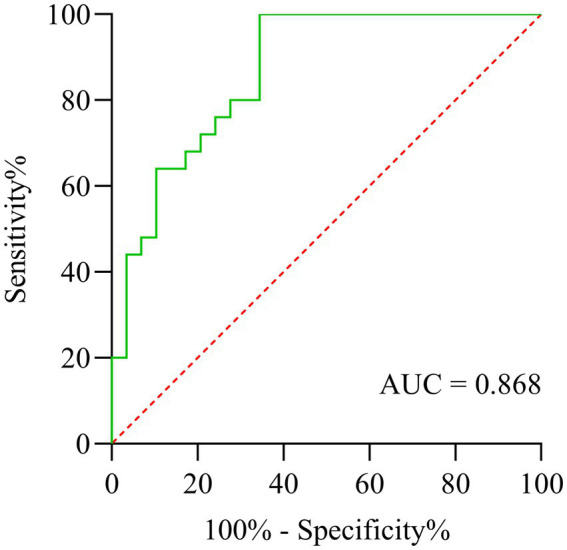
ROC curve analysis of DTI-ALPS index for discriminating cognitive impairment in subcortical infarction patients.

The receiver operating characteristic (ROC) curve analysis (Figure 5) revealed that the DTI-ALPS index showed significant discriminative capacity in identifying cognitive impairment among subcortical infarct patients, achieving an area under the curve (AUC) of 0.868 (95% CI: 0.77–0.96). While demonstrating excellent sensitivity at 96.0%, the specificity remained limited to 65.5%, suggesting potential utility as a screening tool despite its moderate false-positive rate.

## 4 Discussion

This is a positive result in our study that the DTI-AIPS index of subcortical infarct patients was significantly lower than that of HC group, suggesting that the changes of cerebral glymphatic function in patients with stroke. Meanwhile, the DTI-ALPS index on the lesion side was correlated with the MoCA score of day-7 post-stroke, indicating that the DTI-ALPS index was associated with early cognitive function changes. This study found a correlation between early altered in the GS and cognitive impairment after stroke, suggesting that the GS may play an important role in the early changes in cognition function following a stroke. Active interventions targeting GS function in the early stages may provide effective evidence for the prevention and treatment of PSCI.

The energy depletion of oligodendrocytes caused by cerebral ischemia–reperfusion injury leads to demyelination, axonal degeneration and further apoptosis of neurons on the occurrence of cerebral infarction (25, 26). AQP4 water channels in the foot processes of perivascular astrocytes facilitate water movement from the vascular space across the BBB into cells. The glymphatic system is a highly organized fluid transport system that is capable of removing brain waste, regulating the secretion of neurotransmitters and keeping up neuronal function (27). Damaged GS has been found in AD, hydrocephalus, PD, age-related iron deposits as well as tumor-associated brain edema (16). Iliff et al. (20) found that the AQP-4 gene knockout mice have the significantly decreased influx and clearance efficiency on GS than in controls. Further, Lin et al. (28) hold that the perivascular AQP-4 depolarization and intracranial hypertension after ischemic stroke, leading to increased resistance of ISF outflow pathway, great changes of the clearance efficiency have been made in GS, caused by mainly involving the abnormal protein deposition, may be related to vascular dementia (29).

Studies have shown that age-related cognitive decline is associated with the decreased ability of the GS to clear Aβ (30, 31). At the same time, Eide et al. (32) found that the decrease of clearance efficiency of GS leads to the increase of soluble Aβ and tau protein accumulation in the entorhinal cortex-hippocampal circuit, which promotes the development of dementia in patients with idiopathic hydrocephalus. During the acute phase of stroke, pathological changes such as inflammatory stimulation and oxidative stress lead to a reduction in the clearance of harmful substances by lymphocytes. Our research has found that there is glymphatic dysfunction in acute stroke, which is consistent with other studies (33). It is known that the clearance of Aβ drained through the ISF. Dysfunction of the GS leads to an excess of free Aβ, which accumulates in the brain parenchyma and vascular system, promoting the onset of cognitive impairment (34). We found that alterations in the GS are associated with cognitive decline at 7 days post-stroke. Furthermore, we observed significant correlations between 90 days post-stroke MoCA scores and ALPS index, which were consistently demonstrated in the lesion, non-lesion, and mean bilateral measurements. A potential mechanistic explanation is that the enrolled stroke patients exhibited prevalent features of cerebral small vessel disease with white matter hyperintensities and cerebral microbleeds, and these chronic vascular pathologies may exert more profound effects on cognitive function at 90 days than the acute infarct lesions per se. Our ROC analysis demonstrated that the DTI-ALPS index showed moderate discriminative ability (AUC = 0.868) in differentiating patients with cognitive impairment from those with normal cognition following subcortical infarction, exhibiting excellent sensitivity (96.0%) but suboptimal specificity (65.5%). Although these findings suggest the potential clinical utility of DTI-ALPS as a supportive biomarker, its current specificity limitations preclude its recommendation as a standalone screening tool for early detection of post-infarction cognitive impairment. CSF Aβ42, p-tau181 and several plasma p-tau biomarkers (such as p-tau 231) can be used in a specialized memory clinic as a stand-alone biomarker to detect biologically-defined AD (35, 36). The combination of ALPS index and CSF p-tau levels may serve as a stronger predictor of post-stroke cognitive function.

The DTI-ALPS method assesses glymphatic system function by measuring the direction of water molecule movement within the PVS. The ALPS index is calculated for a ROI to quantify GS function. Impaired GS function is represented by a decrease in the DTI-ALPS index (12). DTI-ALPS has been widely utilized to evaluate alterations in the GS associated with neurological and other systemic disorders, such as traumatic brain injury, stroke, carotid atherosclerosis, CSVD, sleep disorders, AD, PD, acute lymphoblastic leukemia in children, and type 2 diabetes mellitus. The largest DTI-ALPS study to date, which included 2,219 participants, found that GS dysfunction, as reflected by DTI-ALPS, is closely related to the occurrence and severity of CSVD. Moreover, the ALPS index demonstrated superior predictive performance for the presence of CSVD among all eligible residents (37). Additionally, the ALPS index shows a significant negative correlation with Aβ and tau protein deposition and a positive correlation with cognitive scores. The ALPS index mediates cognitive dysfunction associated with Aβ and tau deposition (38). Our previous research also found a decrease in the ALPS index, indicating GS dysfunction, in middle-aged and elderly patients with chronic insomnia, which is associated with cognitive impairment (39).

Our study found that the reduction of ALPS in the side of cerebral lesion was not associated with cognitive dysfunction 3 months after stroke, and may represent the compensatory capacity of the GS. Inadequate clearance of GS plays an important role in the pathophysiological mechanisms of cognitive decline after early stroke. It is a therapeutic target to improve early cognitive impairment by actively improving oxidative stress, inflammatory response, reducing apoptosis and maintaining lymphatic system function after stroke. The relative contribution and causal interaction of the GS in cognitive deficits induced by cerebral infarction warrant further study.

At the same time, this study has several limitations that warrant cautious interpretation of the results. The relatively small sample size and single-center recruitment strategy may have reduced statistical power for correlation analysis. While our model showed acceptable discrimination (AUC = 0.868, 95% CI 0.77–0.96), the wide confidence intervals reflect limited precision due to sample size constraints. Second, although we controlled for vascular risk factors and education level, other premorbid cognitive reserve proxies (e.g., occupational complexity, leisure activities) were not systematically collected. Future studies should incorporate comprehensive cognitive reserve assessments using standardized tools like the Cognitive Reserve Index Questionnaire (CRIq). Third, due to clinical constraints, we did not acquire follow-up MRI scans at 90 days post-stroke. Consequently, our analysis could not evaluate temporal changes in glymphatic function (ALPS index) at the same anatomical locations over time. Future studies should incorporate longitudinal MRI assessments at 90-day timepoints to enable analysis of both glymphatic changes at identical anatomical locations and their relationship with 90-day cognitive outcomes.

## 5 Conclusion

In conclusion, lymphatic system dysfunction is present in cerebral infarction, and it is positively correlated with early cognitive decline after stroke. The DTI-ALPS index’s predictive value at 7 days post-stroke highlights a therapeutic window for interventions targeting glymphatic clearance. In the future, it is important to conduct the correlation between cerebral lymphoid dysfunction in patients with cerebral infarction and clinical function based on multimodal MRI technology.

## Data Availability

The raw data supporting the conclusions of this article will be made available by the authors, without undue reservation.
